# Acute Disseminated Encephalomyelitis (ADEM): Current View into Etiopathogenesis and Clinical Features

**DOI:** 10.3390/brainsci16020201

**Published:** 2026-02-09

**Authors:** Klara Ferenc, Piotr Semik, Justyna Paprocka

**Affiliations:** Pediatric Neurology Department, Faculty of Medical Sciences, Medical University of Silesia, 40-752 Katowice, Poland; s87943@365.sum.edu.pl (K.F.); s83117@365.sum.edu.pl (P.S.)

**Keywords:** acute disseminated encephalomyelitis, ADEM, immune-mediated demyelination, neuroinflammation, molecular mimicry, pediatric neurology

## Abstract

Acute disseminated encephalomyelitis (ADEM) is a rare, immune-mediated demyelinating disorder of the central nervous system (CNS) that predominantly affects children and young adults. ADEM typically follows an infectious or, less commonly, immunization-related trigger, and despite decades of clinical observation, its etiopathogenesis remains only partially understood. Clinically, the diagnosis of ADEM continues to pose significant challenges due to the absence of disease-specific biomarkers and its clinical and radiological overlap with other acquired demyelinating syndromes. This narrative review aims to summarize and critically discuss current knowledge on ADEM, with particular emphasis on its etiopathogenesis and clinical characteristics, highlighting the potential implications of recent research for clinical practice and management of this disease. Particular emphasis is placed on post-infectious immune mechanisms, including molecular mimicry, blood–brain barrier (BBB) disruption, loss of immune tolerance, and neuroinflammatory cascades. A wide spectrum of infectious triggers—viral, bacterial, parasitic—as well as post-vaccination, post-transplantation, paraneoplastic, metabolic, and host-related genetic factors are discussed in the context of immune dysregulation leading to CNS demyelination. We also highlight characteristic clinical and neuroimaging features that may aid in differentiating ADEM from other demyelinating syndromes, while acknowledging current diagnostic limitations. The integration of recent advances in ADEM immunopathogenesis with established clinical and radiological insights underscores the complexity of this disorder and highlights the evolving nature of current concepts regarding its diagnosis and clinical heterogeneity.

## 1. Introduction

Acute disseminated encephalomyelitis (ADEM), also known as post-infectious or immune-mediated encephalomyelitis, is a relatively rare neurological disorder that usually affects children and young adults. It is mainly characterized by an acute immune-driven demyelinating process, involving multiple regions of the brain and the spinal cord [1]. Although ADEM has been recognized for decades, its clinical definition, immunopathological mechanisms, and diagnostic boundaries continue to evolve, particularly in light of recent advances in neuroimmunology and neuroimaging.

In most patients, ADEM develops within 1–2 weeks following a viral or bacterial infection. Other recognized triggers include certain vaccinations, as well as parasitic and fungal infections, though a proportion of cases remain idiopathic [2,3]. However, over the past few years, the range of reported triggering and predisposing factors has expanded, and an increasing number of atypical and unusual clinical presentations has challenged traditional diagnostic frameworks and underscored the clinical heterogeneity of ADEM.

The exact pathophysiological mechanisms underlying ADEM are not yet fully established. However, one of the most widely supported hypotheses, based on current evidence, can be framed as immune-mediated injury to the central nervous system (CNS), probably initiated by molecular mimicry or other dysregulated immune responses in genetically or immunologically susceptible individuals [4].

Clinically, about 70% of ADEM cases have a monophasic course. However, multiphasic variants have also been described, featuring recurrent episodes separated by at least three months [4,5]. ADEM is typically characterized by an acute onset of multifocal neurological deficits along with encephalopathy. When diagnosed early and treated appropriately, outcomes are generally favorable. However, diagnosis poses many challenges, particularly in the context of increasingly recognized atypical presentations. In the absence of disease-specific biomarkers and with uncertainties in its etiopathogenesis, it is often diagnosed late, thus delaying the therapy, which can adversely impact prognosis and increase the risk of severe outcomes [6]. Recent studies have provided additional insights into the disease course and long-term outcomes and have suggested new diagnostic approaches and potential candidate biomarkers, although their clinical applicability is a subject of debate and requires further evaluation.

In this review, we aim to provide an updated and integrated overview of the current understanding of ADEM, with a focus on etiopathogenesis, clinical presentation, and diagnostic challenges. By incorporating recent advances in neuroimmunology and neuroimaging, we aim to delineate how new insights influence disease recognition and differential diagnosis, as well as highlight persisting uncertainties and gaps that warrant further investigation.

## 2. Materials and Methods

This article was conducted as a narrative review aimed at providing a qualitative synthesis of the existing literature on ADEM. A literature search was conducted in PubMed, Medline, and Google Scholar databases using controlled vocabulary and keywords. The main search strategy included the search for “ADEM” or “acute disseminated encephalomyelitis” in combination with related terms, such as “etiology”, “epidemiology”, “pathogenesis”, “symptoms”, or “diagnosis”. To narrow the search outcome, the following filters were applied: including studies only in English and preferably published within the last 10 years. Furthermore, this process excluded studies that did not address the primary search concepts or those written in languages other than English. Each of the databases was subjected to a search independently; the search terms were consistently applied in a line-by-line manner and replicated on all platforms. The initial search period spanned from November 2024 to June 2025, followed by regular updates to incorporate the newly published literature. Titles, abstracts, and full-text articles were assessed for relevance to the scope of the article by two independent reviewers. In addition, a manual search of reference lists and citation tracking was performed to identify further eligible studies. Final inclusion decisions were made collaboratively, and any disagreement between the two reviewers was resolved through discussion. The selected studies were categorized thematically on aspects of ADEM—e.g., etiology, pathogenesis, diagnosis—to enable a structured narrative synthesis of the literature.

## 3. Epidemiology

ADEM is considered a rare disease, with its prevalence varying across populations. Population-based studies have estimated the annual incidence to range from 0.07 to 0.60 per 100,000 individuals per year [7,8,9]. Although ADEM can occur at any age, it most commonly affects children under 10 years, with the peak age of onset reported between 3 and 7 years, showing a slight male predominance with a male-to-female ratio of 1.8:1 [1,10]. No specific ethnic predisposition has been clearly identified across studies [11]. However, regional differences have been described: studies from Asia report higher pediatric incidence rates (approximately 0.47–0.64 per 100,000 children) [8,9], whereas studies conducted in Europe and the United States have found lower incidence estimates (approximately 0.07–0.30 per 100,000) [7,12,13]. Epidemiological data for adult ADEM remain limited and heterogeneous, in part because of variable age at presentation and diagnostic overlap with other demyelinating disorders [11].

ADEM also exhibits a seasonal distribution, with cases occurring more frequently in winter or spring, often following an upper respiratory tract infection [14]. Despite its potential severity, most patients experience complete recovery. Nevertheless, recent meta-analyses indicate that outcomes in adults are worse than those in children, and the overall mortality rate has been estimated at 7.8% (95% CI = 3.3–13.5), and the risk of residual deficits was 47.5% (95% CI = 31.8–63.4) in patients [15].

## 4. Etiology

**Figure 1:**
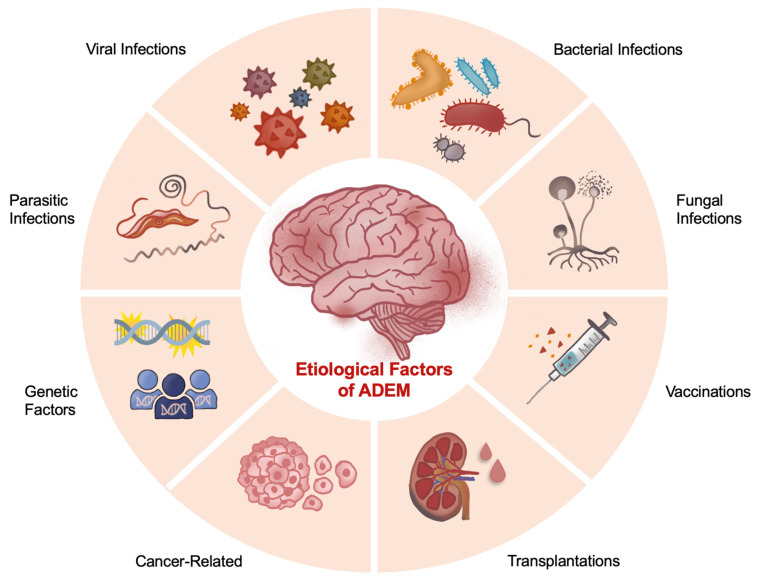
Major etiological factors that have been associated with ADEM.

The etiology of ADEM remains multifactorial and has been linked with a wide array of etiological agents, including both internal and external factors (summarized in Figure 1). In this section, we will discuss the most important etiological factors that have been associated with ADEM across the years.

### 4.1. Viral Infections

ADEM is predominantly considered a post-infectious disease of the CNS. Viral infections, especially infections of the gastrointestinal or respiratory tracts, constitute the most common antecedent event in the etiopathogenesis of ADEM, especially in children. The majority of reports reveal that 50–75% of pediatric ADEM cases are preceded by a viral illness [1,16], and among the most frequently implicated pathogens are measles, mumps, rubella, varicella-zoster virus, influenza virus, Epstein–Barr virus, coronavirus, cytomegalovirus, herpes simplex virus, enteroviruses, and hepatitis viruses [2,3]. However, cases in which ADEM was triggered by Chikungunya and Coxsackie B virus infection have also been reported [17,18]. Moreover, certain viruses were associated with virus-specific features in the clinical presentation of ADEM, which are briefly described in Table 1.

### 4.2. Bacterial Infections

Although viral pathogens constitute the predominant triggers of ADEM, a variety of bacterial agents have also been implicated. Among them, *Mycoplasma pneumoniae* seems to be the most frequent bacterial infection associated with ADEM [45,46]. Beyond *M. pneumoniae*, other bacterial organisms have been reported in association with ADEM, including *Streptococcus pyogenes* and other β-hemolytic streptococci, *Haemophilus influenzae* type b, *Chlamydia pneumoniae*, *Borrelia burgdorferi*, and *Leptospira* spp. In addition, rare cases have linked *Legionella pneumoniae* and *Rickettsia* spp. to subsequent demyelinating syndromes resembling ADEM [2,3]. There are also reports of ADEM occurring after urinary tract infection by *Escherichia coli*, and after gastroenteritis by *Campylobacter* spp. [47,48]. Although rare, *Mycobacterium tuberculosis* has also been described as a potential trigger, and both pulmonary and extrapulmonary forms, including mediastinal tuberculosis in infants, have been followed by ADEM-typical acute demyelinating processes [49].

### 4.3. Parasitic Infections

Cases of parasitic infections have also been linked to the development of demyelinating disorders, including ADEM. One example is infection with *Toxoplasma gondii*, which may represent a relevant cause of ADEM and should be considered, particularly in endemic regions with low socioeconomic conditions, as many *T. gondii* infections remain asymptomatic [50]. Malaria has similarly been implicated in post-infectious demyelination. Infections with *Plasmodium falciparum* have been associated with the onset of ADEM, typically occurring several weeks after antiparasitic therapy, with an average latency of approximately 43–45 days [51]. Although rare, *Plasmodium vivax* has also been reported as a trigger in children. Some reports suggest that the recurrence or new onset of neurological manifestations in patients with a history of severe malaria should raise suspicion for post-malarial ADEM [52,53]. However, the clinical entity known as post-malaria neurological syndrome (PMNS) further complicates this association. PMNS is an uncommon complication occurring one to two months after recovery from malaria. It manifests with acute neuropsychiatric symptoms, but distinguishing PMNS from ADEM can be challenging, because these may represent overlapping or related post-infectious immune-mediated disorders [52].

### 4.4. Post-Vaccinations

Throughout the years, the association between vaccinations and ADEM has been a subject of debate. The incidence of reported post-vaccination cases of ADEM is generally low and it is estimated at approximately 0.1–0.2 per 100,000 vaccinated individuals, accounting for less than 5% of all ADEM cases [54,55]. Post-vaccination ADEM is thought to result from an aberrant immune response to vaccine components, which may be influenced by the target infectious agent of the vaccine, individual immune characteristics of the recipient, the type and amount of adjuvant used, and other factors. The majority of post-immunization ADEM cases are associated with the primary vaccine dose rather than with subsequent doses. The onset of ADEM symptoms typically occurs 8–21 days after immunization, in both adults and children, with the most frequent age of onset in the pediatric population being around 5–7 years in most countries [55].

Historically, ADEM has been associated with vaccines, such as those against influenza, diphtheria–tetanus–polio, human papillomavirus, pertussis, smallpox, measles-mumps-rubella, hepatitis B, rabies, and Japanese B encephalitis [2]. Rare cases of ADEM have also been associated with SARS-CoV-2 vaccination, mainly after the first vaccine dose [56,57]. However, based on current evidence, it remains unclear whether ADEM could be an actual complication of COVID-19, and for sure, this rare condition should certainly not discourage the mass vaccination programs [57]. It is also noteworthy that neurological complications of COVID-19 occur far more frequently after SARS-CoV-2 infection than after vaccination [58].

Furthermore, recent analyses have demonstrated no statistically significant increase in ADEM risk following any vaccine except for the Tdap (adult and adolescent tetanus, reduced diphtheria, acellular pertussis) vaccine, with an estimated excess risk of 0.385 (95% CI = −0.04 to 1.16) cases per 1,000,000 administered doses [54]. Similarly, the study by Chen et al. found no increase in the risk of ADEM or its recurrence in either pediatric or adult populations after vaccination against influenza, hepatitis A or B, polio, diphtheria, pertussis, tetanus, measles, mumps, rubella, Japanese Encephalitis, meningitis, varicella, or rabies. No associations between vaccines and ADEM were observed within 180 days post-vaccination, except for a 31–60-day exposure interval in the pediatric population, which was likely coincidental and not confirmed in separate self-controlled analyses [59].

Therefore, ADEM related to the immunization process should be diagnosed only after exclusion of other potential causes such as viral infection, sepsis, meningitis, multiple sclerosis (MS), or Guillain-Barré syndrome [55].

### 4.5. Post-Transplantations

Other infrequent causes of ADEM are related to organ and hematopoietic stem cell transplantation. Reported cases include ADEM occurring after human leukocyte antigen (HLA)-identical sibling allogeneic bone marrow transplantation (BMT) for transfusion-dependent pure red cell aplasia [60], as well as in children with other hematological malignancies, including acute lymphoblastic leukemia (ALL), acute myeloid leukemia (AML), and chronic lymphocytic leukemia (CLL) [61,62]. Demyelinating syndromes such as ADEM have also been described as late sequelae of autologous peripheral blood stem cell transplant (PBSCT) for non-Hodgkin’s lymphoma, and following BMT for AML in adults [63,64]. Furthermore, although rare, ADEM has been reported in liver and renal transplant recipients. Post-transplant ADEM is thought to result from immune dysregulation, where exposure to donor-derived antigens or opportunistic infections in an immunocompromised host may elicit an aberrant T cell-mediated autoimmune response against myelin components, leading to demyelinating syndromes such as ADEM [65,66].

### 4.6. Other Causes

Rare cases of ADEM have also been reported in association with chemo-radiotherapy for nasopharyngeal cancer, which may potentially damage brain tissue, induce neurotoxicity and, when combined with infection, trigger an immune-inflammatory response leading to subsequent demyelination [67]. In addition, paraneoplastic mechanisms represent another uncommon but recognized cause of ADEM. Paraneoplastic ADEM has been documented in association with several malignancies, including multiple myeloma, small-cell lung carcinoma, and hepatic epithelioid hemangioendothelioma [68,69,70]. Therefore, ADEM should also be considered in cancer patients presenting with new neurological symptoms and demyelinating lesions on neuroimaging.

Recently, a causal relationship between ADEM and folate deficiency has been suggested [71]. Moreover, sporadic case reports have also linked the occurrence of ADEM to exposure to various psychoactive or herbal substances. A case linked to ADEM with chronic cannabis abuse has been described, suggesting that prolonged neurotoxic or immune-modulatory effects of cannabinoids might contribute to demyelination in susceptible individuals [72]. ADEM has been reported following the use of oral or parenteral herbal preparations containing multiple plant extracts such as *Echinacea*, *Aconitum*, or *Adonis vernalis* [73,74].

Moreover, accumulating evidence indicates that the host’s genetic background, especially polymorphisms within the HLA, may play an important role in modulating individual susceptibility to ADEM [18]. For instance, it was found that HLA-DRB1*1501, DRB1*1503, DQB1*0602, DRB1*01, and DRB1*017(03) alleles were significantly associated with predisposition to clinical presentation of ADEM [18,75,76].

## 5. Pathogenesis

Although ADEM is a clinical entity that has been recognized for years, its pathogenic mechanisms are still not fully understood. Currently, several potential mechanisms have been proposed to explain the molecular and clinical features of this disease. Here, we present insights into a few of these pathomechanisms with neuroinflammation appearing to play a pivotal role.

### 5.1. Mechanism of Molecular Mimicry

**Figure 2:**
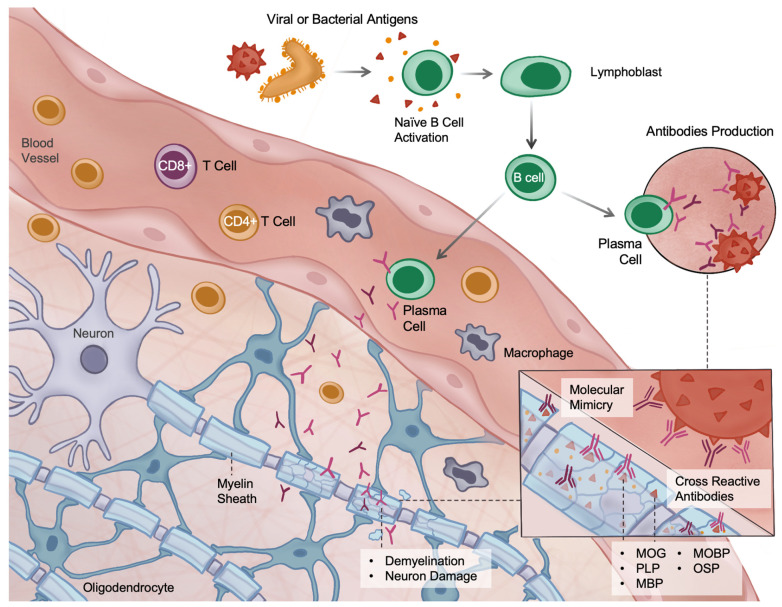
The concept of the molecular mimicry mechanism in ADEM. Activation of naïve B cells following viral or bacterial infection leads to the production of cross-reactive antibodies recognizing both pathogen-derived and myelin antigens, resulting in immune-mediated neuronal injury with involvement of humoral and cellular immune responses. Legend: MOG, myelin oligodendrocyte glycoprotein; PLP, proteolipid protein; MBP, myelin basic protein; MOBP, myelin-associated oligodendrocyte basic protein; OSP, oligodendrocyte-specific protein.

One of the key hypotheses that has been proposed to explain complex ADEM pathogenesis is the molecular mimicry mechanism. Its main concept in ADEM involves molecular similarity between pathogens’ epitopes and autoantigens of the myelin structures, such as myelin oligodendrocyte glycoprotein (MOG), proteolipid protein (PLP), myelin basic protein (MBP), myelin-associated oligodendrocyte basic protein (MOBP), and oligodendrocyte-specific protein (OSP) [11,77]. This similarity results in the induction of autoimmune antibodies production against these structures, which is considered the most significant mechanism causing immune-mediated injury in ADEM, leading to the activation of both humoral (antibody-mediated) and cellular immune response [78] that is presented in Figure 2.

Among these antibodies, anti-MOG antibodies are considered the most commonly occurring in ADEM patients, especially in younger individuals; thus frequency of their occurrence decreases with age. They are detectable in both the serum and cerebrospinal fluid (CSF), especially during the acute stage of the disease, and were found to decrease in the recovery phase [79,80]. The anti-MOG antibodies are predominantly of the IgG1 subclass, and as mentioned before, they can initiate both humoral and cellular immune responses, including taking part in complement activation, leading to CD4-dominant T cell and perivascular macrophages infiltration, and antibody-dependent cell-mediated cytotoxicity (ADCC) [78,81]. The specific target epitope for these antibodies has been identified within the N-terminal, immunoglobulin-like extracellular domain of MOG, specifically residues MOG35–53, which is expressed mainly by mature oligodendrocytes, and associated with their maturation, maintenance of myelin integrity, and facilitation of intercellular adhesion and cellular interactions [11,82].

Moreover, anti-MOG-positive patients have been found to more often develop relapses of ADEM and have an earlier onset of symptoms than seronegative patients. However, anti-MOG antibodies could also be detected in other demyelinating disorders, including recurrent optic neuritis or aquaporin-4-antibody-seronegative neuromyelitis optica spectrum disorder, and cannot be considered specific for ADEM [80].

### 5.2. Post-Infectious Blood–Brain Barrier Disruption

Alternative mechanisms of ADEM pathogenesis involve CNS tissue injury and blood–brain barrier (BBB) disruption, which may result from direct infection of the CNS by neurotropic pathogens. Such infections can cause the release of CNS-restricted autoantigens into the peripheral circulation, where they are subsequently processed by immune cells within systemic lymphoid organs. This process may contribute to the loss of self-tolerance and the development of autoreactive, encephalitogenic T cell responses targeting CNS components, resulting in characteristics for ADEM-disseminated demyelination areas in the brain and spinal cord [83].

**Figure 3:**
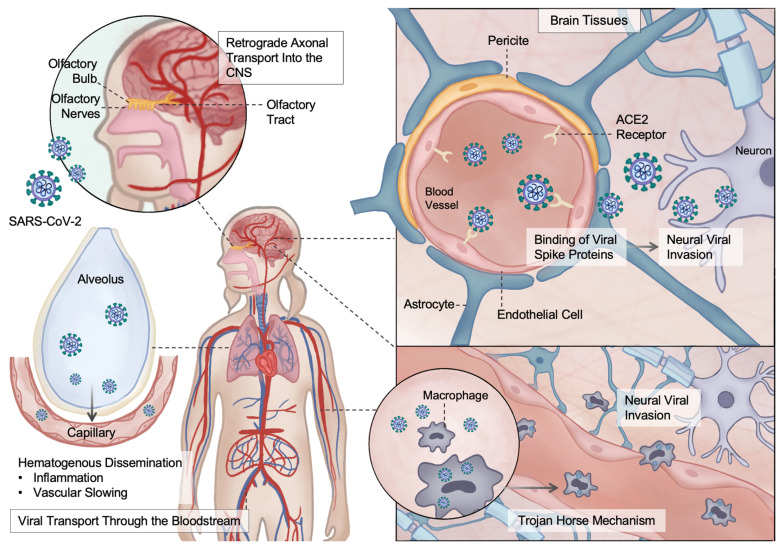
Proposed pathways of ADEM initiation following SARS-CoV-2 infection. Potential mechanisms include hematogenous spread facilitated by systemic inflammation, retrograde axonal transport via olfactory nerves, transendothelial passage across the blood–brain barrier (BBB) mediated by ACE2 receptor binding, and leukocyte-mediated CNS entry (Trojan horse mechanism). Legend: ACE2, angiotensin-converting enzyme 2; CNS, central nervous system.

Such pathogen-induced mechanisms have been described for several neurotropic viruses, including SARS-CoV-2. As a neuroinvasive virus, SARS-CoV-2 is capable of penetrating the CNS through multiple routes (Figure 3). These include: (i) hematogenous dissemination with viral transport through the bloodstream and subsequent neural invasion, facilitated by inflammation-induced vascular slowing; (ii) migration of infected leukocytes across the nerve tissue through the glial-lymphatic system in a process referred to as the “Trojan horse” mechanism; (iii) direct binding of viral spike proteins to angiotensin-converting enzyme 2 (ACE2) receptors located on endothelial cells of the BBB or epithelial cells of the blood–CSF barrier at the choroid plexus; and (iv) retrograde axonal transport along peripheral olfactory neurons directly into the CNS [33,84,85,86,87]. Through these pathways, SARS-CoV-2 may contribute to BBB disruption, exposure of CNS antigens, and subsequent immune dysregulation resembling the post-infectious mechanisms proposed in ADEM.

### 5.3. Neuroinflammation Processes

Regardless of the initiating mechanism, neuroinflammation seems to play a central role in ADEM pathogenesis. One of the most important events in the ADEM progression is increased releasement of cytokines and chemokines, associated with activation of certain inflammatory cascades, and resulting in immune-mediated neuronal damage. The elevated levels of cytokines and chemokines are commonly found in the CSF and serum of ADEM patients, revealing the presence of cytokine-mediated responses in ADEM [88]. However, its profile depends on stage of the disease.

During the acute phase of illness, T-1 helper cells and their cytokines are considered the principal agents. The excessive activation of these immune cells results in increased levels of certain cytokines, such as interleukin-1β (IL-1β), IL-6, IL-8, tumor necrosis factor-α (TNF-α), or interferon-γ (IFN-γ). Moreover, the marked elevation in the E-selectin level was also observed and correlated with the early phase of lymphocyte migration across the BBB in ADEM [89,90,91]. Furthermore, the elevated levels of various adhesion molecules, including intercellular adhesion molecule-1 (ICAM-1), matrix metalloproteinase-9 (MMP-9), and tissue metallopeptidase inhibitor-1 (TIMP-1) were also noticed, and could be considered as markers of BBB disruption [88,90,92,93].

On the other hand, during the stage of clinical remission, there is a shift to T-2 helper cells dominance, resulting in increased levels of IL-4, IL-10, and transforming growth factor-β (TGF-β). This phase is also associated with downregulation of E-selectin and ICAM-1, and enhanced expression of vascular cell-adhesion molecule-1 (VCAM-1). Additionally, elevated concentration of IL-12 and increased stimulation of IFN-γ-producing CD4 + memory T cells were also observed in the ADEM remission stage [2,90,94].

Altogether, these processes are leading to immune-mediated neuronal injury and varying in contribution to these cellular activation patterns; this mainly involves T lymphocytes, monocytes/macrophages, and occasionally neutrophils, which have been reported in inflammatory infiltration of white matter in ADEM [18]. Additionally, the axonal damage in ADEM is also indicated by the increased level of phosphorylated Tau (p-Tau) protein, which is the microtubule-associated protein, and its elevated presence in the CSF is correlated with the clinical severity of ADEM [95].

## 6. Diagnosis

### 6.1. Clinical Features

Pediatric ADEM belongs to a group of illnesses distinguished by an acute or subacute onset of neurological deficits associated with evidence of inflammatory demyelination of the CNS, including the optic nerves. That group of conditions is known as acquired demyelinating syndromes (ADSs). ADEM is a common subtype of ADS and includes about 22–32% of children with ADS [2,3,11,96,97].

The diagnosis of pediatric ADEM requires a mix of clinical features and magnetic resonance imaging (MRI) findings. Encephalopathy is an obligatory feature and includes behavioral changes and modification of consciousness, not explained by systemic febrile illness or postictal symptoms [4,98,99,100,101]. According to the latest (2013) International Pediatric Multiple Sclerosis Society Group (IPMSSG) standards, ADEM should be suspected among children who suffer from polyfocal neurological deficits with encephalopathy. It is important to prove the lack of impact of any previous neurological disorders and to collect a history of past viral infections or vaccinations. Crucially, other conditions, such as viral or bacterial encephalitis, must be eliminated before a definitive diagnosis can be established [3,4,102]. Table 2 presents the most common symptoms of ADEM in children and their frequency of occurrence.

Since there can be variations in the progression of the disease during the first three months, recurrence of symptoms, or onset of new symptoms within this time frame, are considered part of the initial ADEM event. If a second episode, which can be defined by new or prior manifestations or MRI lesions (and qualifies as ADEM), happens after three months, the designation “multiphasic ADEM” is applied. Individuals in the multiphasic group more frequently develop a clinical flare, as opposed to monophasic patients [3,4,106]

Diagnosing ADEM proves to be complex, given the absence of specific biomarkers confirming the diagnosis and the variability in clinical presentations. No laboratory indicators or procedures can establish a conclusive diagnosis, although various examinations—MRI, CSF, electroencephalogram (EEG)—may be helpful [3,11]

**Figure 4:**
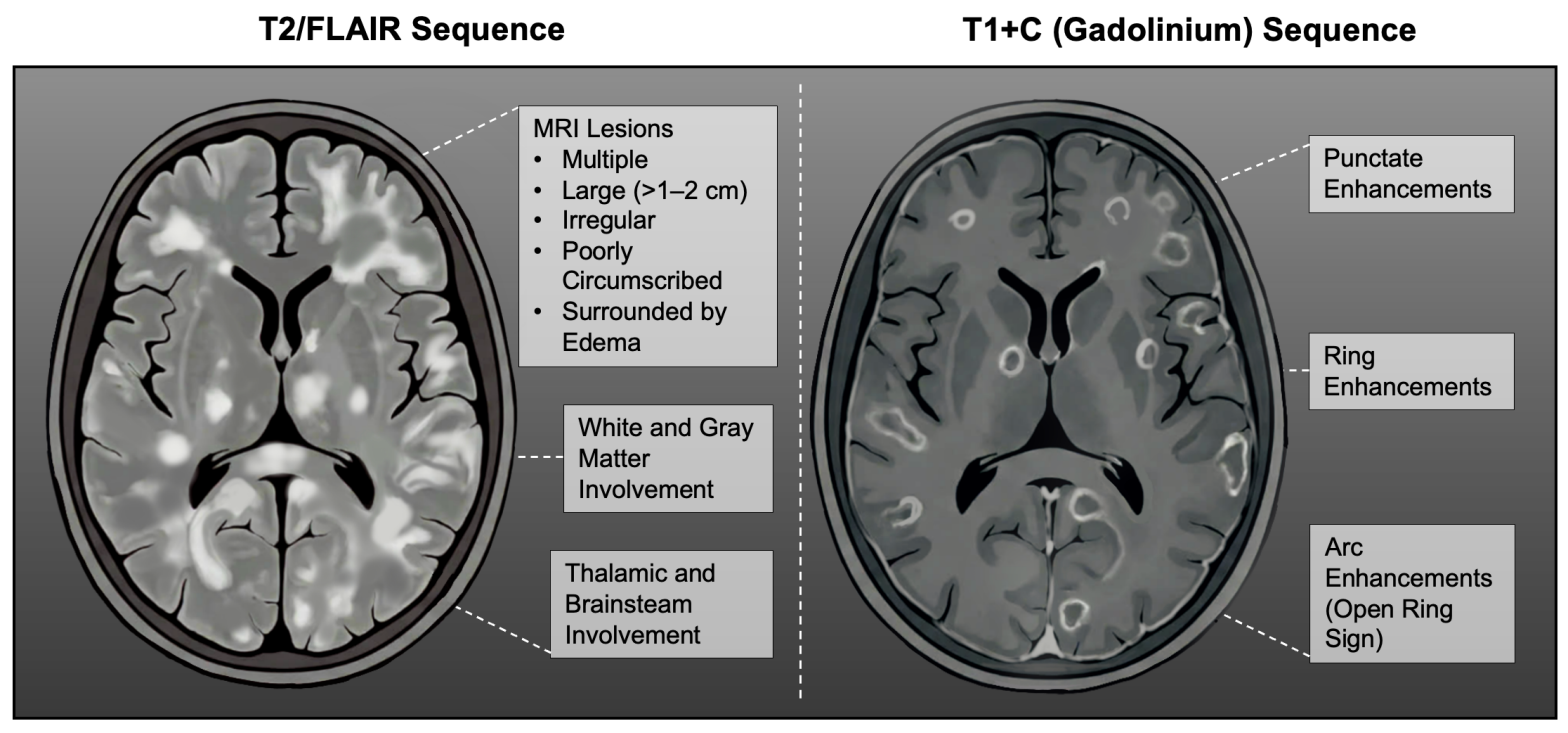
Typical MRI changes in ADEM visible in T2/FLAIR and T1+C (Gadolinium) sequences.

MRI is regarded as the most effective modality for confirming the presence of ADEM and excluding other potential causes of demyelination in the central nervous system, including conditions like MS or neuromyelitis optica, which may mimic ADEM in their clinical manifestation. Lesions are commonly seen on T2-weighted and FLAIR scans as multiple, variably sized, and irregular regions (Figure 4) [2,3]. MRI can demonstrate abnormalities dispersed across both white and gray matter regions of the brain. The subcortical and central white matter of the entire CNS (including the brainstem and spinal cord) is commonly affected, with the frontal and temporal lobes being particularly involved. Regarding gray matter, the thalamus and basal ganglia may be affected, though lesions are most frequently observed at the junction between cortical gray matter and underlying white matter [2,102]. Compared to MOG-IgG seronegative ADEM cases, children with seropositive MOG-IgG ADEM were more likely to have lesions located in the cortex and thalamus. T1-weighted imaging usually does not reveal these lesions, though larger ones may appear as hypointensive areas. On early follow-up imaging, multiphasic ADEM patients demonstrated new lesions more frequently than monophasic individuals [102,106,107]. Spine MRI explains the cause of sensory changes, extremity muscle weakness, and bowel and bladder dysfunction. Spinal cord lesions can be varied, short and limited to two vertebral segments, or extensive, extending to three or more vertebral segments. On imaging, ADEM-related lesions generally appear with blurred or indistinct edges, which may help differentiate them from MS lesions that typically demonstrate sharp, well-delineated edges. Occasionally, an ADEM patient’s MRI may appear with no abnormalities. In some cases, radiological changes may emerge weeks after the onset of clinical signs [100,102,108].

Examination of CSF, when ADEM is suspected, is mainly used to rule out meningoencephalitis or viral CSF infections, which can present with similar symptoms. CSF findings may range from normal to mildly increased white blood cells level, predominantly lymphocytic and monocytic. Usually, there is also increased protein level or, according to IPMSSG consensus—on rare occasions, transient oligoclonal bands, as confirmed by the study carried out by Boudjani et al. involving 45 patients (adults and children) with myelin oligodendrocyte glycoprotein antibody-associated disease (MOGAD) [109], where oligoclonal bands were not observed in 87% of cases (26/30 lumbar punctions) [2,3,96,109].

EEG can serve as a valuable tool in guiding the diagnostic process. In viral encephalitis, it often reveals non-specific background slowing. Although periodic lateralized epileptiform discharges (PLEDs) may suggest herpes simplex encephalitis (HSE), this finding is neither highly sensitive nor specific. In pediatric patients presenting with unexplained disturbances of consciousness, an EEG is indicated to rule out non-convulsive status epilepticus. In addition, the presence of extreme delta brush patterns may be indicative of underlying anti-NMDA receptor encephalitis [78,98,101].

### 6.2. Differential Diagnosis

Differential diagnosis in ADEM contains other autoimmune demyelinating disorders, genetic and metabolic diseases, stroke-like episodes, CNS vasculitis, lymphoma, histiocytosis, anti-NMDAR encephalitis, or systemic lupus erythematosus [4,96,110,111].

In older children and adolescents, we have to exclude autoimmune conditions by serological tests (such as systemic lupus erythematosus or Hashimoto encephalopathy). A urine toxicology screen also should be performed. In young children with developmental delay and unexplained encephalopathy, it is necessary to rule out metabolic disorders. However, if there is still no clear diagnosis, the brain biopsy ought to be conducted [98,101].

In ADEM, biopsy is generally not required to establish the diagnosis. Nevertheless, in pseudotumoral forms, tissue sampling and histopathological evaluation may be warranted to rule out alternative diagnoses, including other demyelinating diseases, abscess, or tumors. Brain biopsy may also be justified, particularly when the differential diagnosis includes primary CNS angiitis or tumorous lesions that raise suspicion of an underlying neoplastic process [101,112].

To differentiate between changes in the CNS revealed by imaging studies, it may be useful to use proton magnetic resonance spectroscopy (MRS). In the study performed by Ikeguchi et al., results showed that with MRS use, we are able to distinguish tumefactive demyelinating lesions (TDLs) and brain tumors. In cohort nr 1 (six patients with TDLs and five patients with gliomas, including three high-grade) gliomas showed a significantly elevated mean choline/N-acetylaspartate (Cho/NAA) ratio compared with TDLs and MS (*p* < 0.05). Furthermore, across both cohorts (cohort nr 2–6 patients with TDLs and 17 patients with gliomas, including 8 high-grade), high-grade gliomas exhibited a significantly higher mean Cho/NAA ratio than TDLs (*p* < 0.05) [113].

The improvement in diagnostic methods allows us to link the same ADEM cases to the occurrence of MOG antibodies. MOG antibody disease is a rare autoimmune disorder, but it induces encephalitis, encephalopathy, or ADEM-like episodes. Antibodies against the MOG attack the optic nerve and spinal cord, which causes loss of vision and paralysis [78,114,115,116,117,118,119]. When MOG antibody disease takes up the brain, the phenotype is similar to ADEM with a modification in mental status.

Another disease that may be clinically similar to ADEM is MS. ADEM, commonly preceded by an infection or immunization, which tends to follow a single-phase course and generally has a positive outcome. By contrast, MS more often progresses through repeated relapses and remissions, with each episode contributing to incremental neurological dysfunction. Both diseases may present polyfocal neurological deficits and lesions in diagnostic imaging, although some changes are more common for ADEM. To distinguish between them, it is best to use MRI [2,3,120]. Table 3 shows common MRI lesions for ADEM and MS.

Tumefactive ADEM represents an uncommon form of ADEM, distinguished by the presence of large mass—like abnormalities on neuroimaging—that can closely mimic brain tumors on MRI. Unlike typical ADEM, which usually manifests with multiple disseminated lesions, tumefactive ADEM tends to present with solitary or a few large focal abnormalities and a more localized neurological deficit [122]. Table 4 shows a brief summary of the differences between ADEM, tumefactive MS, and Baló’s concentric sclerosis (BCS).

### 6.3. Diagnostic Future

Recent advancements in machine learning show promising potential as powerful tools capable of improving the diagnostic process in both general medicine and neuroscience, with particular relevance to demyelinating diseases. Radiology is already incorporating machine learning tools, and their use is expected to expand quickly in the near future. With the rise of artificial intelligence (AI) and technology, thorough understanding of machine learning tool behavior is crucial for using them safely and effectively [121]. According to a study conducted by Wei et al., machine learning models can effectively differentiate MOGAD ADEM—like the case from standard ADEM based on MRI FLAIR characteristics. A total of 1041 radiomic features were extracted from MRI FLAIR lesions. Redundancy analysis, LASSO (least absolute contraction and selection operator), and significance testing were applied to select candidate predictors. Selected features combined with MOG antibody results were used to train a machine learning classification model. Between April 2015 and January 2020, a retrospective analysis was conducted on 70 patients with ADEM-like presentations. Among them, 49 were diagnosed with classic ADEM and 21 with MOGAD. Clinical data were obtained from the Children’s Hospital of Zhejiang University. The machine learning-based classification achieved an accuracy exceeding 78.6%, with an AUC (area under the curve) above 89% [127].

Achieving high diagnostic precision in recognizing MS and neuromyelitis optica (NMO) can be accomplished through the use of a multi-parametric phenotype approach. Consequently, it appears to be a valuable aid for optimizing diagnostic procedures, enhance accuracy of biomarker variation analysis, and ensuring better differential diagnosis [128,129,130].

Based on a study by Nguyen et al., in ADEM patients, those positive for MOG-IgG (71.1%) more frequently displayed optic nerve involvement, and MRI abnormalities tended to persist. In contrast, MOG-IgG negative children were more prone to complete disappearance of radiological lesions (75.5% vs. 22.2%, *p* < 0.05). Results highlight the importance of MOG-IgG testing, optical coherence tomography (OCT), and specialized orbital imaging as components of the diagnostic process and ongoing follow-up of children with ADEM [131].

Despite the high potential of the proposed diagnostic methods, the limited amount of data and validation on small cohorts prevent these approaches from being relied upon exclusively for the diagnosis of this disease; however, they may provide a valuable direction for the further development of ADEM diagnostics.

## 7. Course of Illness

In the majority of cases, ADEM manifests as a monophasic, self-limiting condition with neurological status typically restoring within three months following disease onset. Usually, symptoms onset 1–2 weeks after infection. Early-phase symptoms, such as fever, headache, nausea, vomiting, and fatigue, are typically noted in affected patients, and during the acute phase, encephalopathy manifests itself as behavioral and conscious disturbances. Due to the fact that the demyelination process may affect various areas of the CNS, symptoms may vary, e.g., if the motor cortex is damaged, we may see signs such as the Babinski sign, paraplegia, spasticity, and hyperreflexia. From the emergence of symptoms to their peak intensity spans approximately 4 to 7 days [3,101].

Although ADEM is typically classified as a monophasic disorder, certain studies have indicated that 25–33% of patients with ADEM may experience a recurrence. Findings from a study performed by Zang et al. suggest that around one in four adults diagnosed with ADEM (56 patients > 18 years old) present a multiphasic form of the disease. Additionally, the study identified that certain early clinical features—particularly optic nerve involvement and autonomic dysfunction—appeared more frequently in patients with multiphasic ADEM [5,132,133,134]. Table 5 presents the main types of ADEM courses and their most common clinical outcomes.

Comparing pediatric and adult ADEM populations remains challenging due to the absence of internationally accepted diagnostic criteria for adult cases. The IPMSSG criteria, which rely on clinical and neuroimaging findings, have shown partial effectiveness in identifying pediatric ADEM. However, due to the lack of alternative criteria for adults, their application to adult patients results in more than half of cases being left without a definitive diagnosis. Despite similar clinical manifestations in pediatric and adult populations, adults often experience a more aggressive course of disease. Older patients, in particular, are prone to multiple complications, higher Intensive Care Unit (ICU) admission rates, less favorable recovery, longer hospitalization, and elevated mortality rates. Compared with children, adults were less likely to present with fever, which may reflect age-associated modifications in the immune system, especially in inflammatory cytokine responses. MRI findings in children more frequently reveal lesions in the thalamus and basal ganglia, while adults more often present with periventricular lesions and increased gadolinium-enhancing lesions (30% vs. 55–60%). Another difference may be impaired consciousness, which is reported in 46–73% of children and 20–56% of adults [4,11,15].

## 8. Overview of the Treatment Approaches

In the absence of randomized studies specifically designed to evaluate the efficacy of treatment in ADEM, current therapeutic approaches are largely based on observational studies and consensus opinions. Initial supportive care and empirical antiviral and antibacterial therapy may be considered in the management of ADEM, as the presentation may mimic infectious encephalitis [1,137].

Accumulating evidence suggests that early recognition and prompt initiation of immunomodulatory treatment may be associated with improved clinical outcomes [16,96,111]. The cornerstone of acute therapy is high-dose intravenous corticosteroids, most commonly methylprednisolone administered at a dosage of 10–30 mg/kg/day (up to a maximum of 1000 mg/day) for 3–5 days, followed by an oral prednisone taper over 4–6 weeks (1–2 mg/kg/day, up to 60 mg per day), or, instead of methylprednisolone, dexamethasone at 1 mg/kg/day for 3–5 days [1,96,138]. Premature withdrawal of corticosteroids, particularly within the first three weeks, has been associated with an increased risk of disease recurrence [139]. During steroid therapy, the careful monitoring of blood pressure, electrolytes, and glucose levels is required, and appropriate gastric protection should be provided [140]. Typically, neurological improvement is observed within days of initiating steroid therapy, with most patients achieving a substantial or complete clinical recovery within a few weeks [141].

In cases where clinical response to corticosteroids is insufficient, intravenous immunoglobulin (IVIG) is commonly employed as second-line therapy, typically administered at a total dose of 2 g/kg over 2–5 days. IVIG is generally well-tolerated in pediatric patients and has demonstrated benefit in steroid-refractory cases [142].

For patients with severe or treatment-resistant disease, therapeutic plasma exchange consisting of three to seven exchanges may be considered [143,144,145]. The efficacy of plasma exchange (PE) has been supported by studies in adults with demyelinating disorders, as well as in pediatric patients with inflammatory CNS diseases, including ADEM, where it is regarded as a safe and effective rescue therapy [143,145,146]. In rare fulminant cases complicated by refractory intracranial hypertension, decompressive craniectomy has been reported as a life-saving intervention when immunotherapy and intensive care measures fail [140].

Clinical improvement is often observed within days following initiation of therapy [96,137], but follow-up MRI is recommended to evaluate disease evolution and to identify potential multiphasic courses [1]. Although the optimal timing of follow-up imaging remains debated, a control MRI performed approximately three months after the acute event is commonly considered reasonable, as radiological findings may fluctuate during the early recovery phase [4].

On the other hand, management of multiphasic ADEM remains particularly challenging. Data from a European collaborative study indicate that disease-modifying therapies typically used in MS, such as interferons or natalizumab, are not associated with clinical benefit in children with MOG-IgG–associated disease, such as ADEM, whereas B–cell–targeted approaches, especially maintenance intravenous immunoglobulin therapy, are linked to a reduced relapse rate [147]. Other reports suggest that prolonged oral corticosteroid treatment may also be associated with a lower risk of relapse. Overall, treatment decisions should be individualized and based on careful clinical and radiological follow-up, and further prospective studies are needed to define optimal therapeutic strategies and long-term outcomes in this subgroup of patients [4].

## 9. Conclusions

ADEM remains a clinically and biologically heterogeneous immune-mediated demyelinating disorder, characterized by variable clinical and radiological presentation. The evidence summarized in this review indicates that ADEM cannot be explained by a single pathogenic mechanism, but rather reflects a complex interplay between environmental triggers, host susceptibility, and CNS-specific factors. Despite advances in our current understanding of these processes, certain aspects of the initiation and evolution of the disease remain to be defined.

One of the main issues highlighted in the current review is the ongoing difficulty in achieving a timely and confident diagnosis of ADEM, especially in atypical or borderline cases. The overlap with other acquired demyelinating syndromes and the absence of disease-specific laboratory markers continue to limit diagnostic precision, placing MRI and exclusion of alternative causes at the core of accurate diagnosis. Moreover, in consideration of the multiphasic variants of ADEM and its ambiguous presentation, long-term observation is considered to be crucial for diagnostic clarification and accurate prognostic assessment.

In conclusion, ADEM should be viewed as a spectrum of post-inflammatory CNS disorders rather than a uniform clinical entity. A balanced interpretation of clinical, radiological, and immunological data is crucial for optimal patient management. Meanwhile, ongoing research is necessary to address remaining uncertainties in both the clinical and biological aspects of this disease, aiming to further improve and deepen our understanding of patient outcomes.

## Figures and Tables

**Table 1:** Main viral agents implicated in ADEM with their reported and unusual clinical features.

Etiological Agent	Characteristic Features of Viral Infection-Associated ADEM Described in Clinical Reports	Reference
Human Immunodeficiency Virus (HIV)	• May occur during primary HIV infection (seroconversion) or in the setting of mild to moderate immunodeficiency. • Reported to follow a more aggressive and atypical clinical course with multiphasic presentation. • Radiological findings can be atypical and include tumefactive lesions and corpus callosum lesions. • Immune dysregulation in HIV predisposes to co-infections (e.g., Herpesviridae, Cryptococcus neoformans) that may act as additional triggers of ADEM.	[19,20,21,22]
Dengue Virus (DENV)	• Rare complication, with an estimated prevalence of ~0.4% among dengue patients, characterized by a notably higher mortality compared to other viral triggers. • Onset may occur either early or late after DENV infection, including during the convalescence phase and in association with dengue hemorrhagic fever (DHF). • Clinical and radiological features are consistent with typical ADEM, most commonly presenting with altered consciousness, seizures, urinary disturbances, and visual impairment, together with characteristic demyelinating lesions. • Higher fever at admission and an early onset of neurological manifestations are associated with worse outcomes.	[23,24,25]
Measles Virus (MV)	• Uncommon but severe complication of measles infection, reported in both children and adults, with poorer outcomes in adults compared to children, even in otherwise immunocompetent individuals. • Historically associated with high mortality and with 25–40% of survivors developing permanent neurological sequelae.	[26,27,28]
Epstein–Barr Virus (EBV)	• EBV infection has been linked to the induction of anti-myelin oligodendrocyte glycoprotein (MOG) antibody production, predisposing to a more severe, anti-MOG antibody–positive form of ADEM. • Due to the EBV predilection for B cells, clinical improvement has been reported with B cell–depleting therapies such as rituximab.	[29,30,31]
Severe Acute Respiratory Syndrome Coronavirus 2 (SARS-CoV-2)	• Among patients diagnosed with COVID-19 and exhibiting neurological symptoms, 10% of patients had ADEM or ADEM-like lesions. • Shares most features with classical ADEM but differs by a longer latency between infectious prodrome and neurological onset, a tendency to affect older patients, less favorable outcomes with lower rates of complete recovery, more frequent involvement of periventricular white matter and the corpus callosum, and less frequent deep gray matter lesions.	[32,33,34]
Cytomegalovirus (CMV)	• May progress in acute hemorrhagic leucoencephalitis (AHLE) without early diagnosis.	[35]
Herpes Simplex Virus (HSV)	• May present in atypical forms, including transverse myelitis, and is considered a marker of poor prognosis.	[36]
Human Herpesvirus 6 (HHV-6)	• Reported progression from HHV-6 encephalitis to ADEM, even in immunocompetent children. • HHV-6 infection has also been implicated in induction of acute necrotizing encephalopathy with a presence of radiological features resembling an ADEM-like demyelinating pattern.	[37,38,39]
Hepatitis C Virus (HCV)	• Acute CNS demyelination might be the first manifestation of HCV infection.	[40]
Influenza Type A	• May be associated with a recurrent, multiphasic form of ADEM.	[41]
Mumps Virus (MuV)	• May occur following mumps infection even in a fully vaccinated and immunocompetent patients.	[42]
Varicella Zoster Virus (VZV)	• Reported as a rare complication of VZV infection, including cases with unusual longitudinally extensive hyperintense spinal cord lesions. • VZV infection was associated with anti-MOG antibody–positive form of ADEM.	[43,44]

**Table 2:** General symptoms of ADEM in children [3,103,104,105].

Symptoms	Frequency
Encephalopathy	100%
Fever	12–80%
Headaches	6–64%
Bladder Dysfunction	10–61%
Pyramidal Symptoms	18–60%
Ataxia	36–53.5%
Seizures	18.5–50%
Cranial Nerve Dysfunction	6.6–45%
Optic Neuritis	6.6–27%
Sensory Disorders	0–17%

**Table 3:** Comparison of ADEM and MS in MRI features [96,121].

MRI Features	Common For:	
	ADEM	MS
Single Well-Defined Lesions	NO	YES
Lesion Perpendicular to Long Axis of Corpus Callosum	NO	YES
Black Holes	NO	YES
Poorly Circumscribed Lesions	YES	NO
≥1 Gadolinium-Enhancing Lesion	NO	YES
Basal Ganglia/Thalamus	YES	NO
Non-Focal Lesions on Both Sides	YES	NO

**Table 4:** Comparison of ADEM, tumefactive MS, and BCS [102,109,123,124,125,126].

Feature	ADEM	Tumefactive MS	BCS
Definition	Acute immune–mediated demyelinating CNS disorder (often post-infection)	Atypical variant of MS, characterized by large (>2 cm) demyelinating lesions	MS variant (rare demyelinating CSN, concentric layered lesions)
Epidemiology	Mostly children, also adults	Mostly adults (uncommon)	Mainly young adults (rare)
Typical Onset	Acute, often within weeks after a preceding infection	Subacute to acute neurological symptoms	Subacute to acute neurological symptoms (may mimic tumor)
Clinical Presentation	Acute onset with encephalopathy (e.g., confusion), focal neurological deficits, often fever, headache, pyramidal symptoms	Focal deficits depending on lesion location, often mimics a brain tumor	Focal deficits depending on lesion location (e.g., motor weakness), encephalopathy less prominent than in ADEM
MRI Findings	Multiple demyelinating lesions, usually without significant mass effect (exception—tumefactive ADEM), often poorly defined	Up to few large lesions, mass effect, possible edema, may mimic tumors	Characteristic concentric ring lesions—alternating bands of demyelination and preserved myelin
CSF Findings	Often non-specific, mild pleocytosis, elevated protein, oligoclonal bands mostly absent	Usually positive for oligoclonal bands, but less consistently detected than in classic MS	Oligoclonal bands less frequently detected compared with MS, variable findings
Disease Course	Usually monophasic	Usually chronic, with potential relapses	Monophasic or relapsing, may show variable outcomes including progression

**Table 5:** Course of ADEM and typical outcome.

Course of Illness	Clinical Course (Summary)	Prognosis	Reference
**Monophasic ADEM**	• Typically occurring as a single, self-limited episode, frequently following a viral infection or recent vaccination. • Characterized by an acute onset with multifocal neurological sings and cognitive dysfunction. • High-dose corticosteroids are effective in the majority of patients.	• Typically positive, as most patients recover fully. • Mild, lasting neurological deficits may be observed, especially in adults.	[4]
**Multiphasic ADEM**	• Marked by a new demyelinating relapse with new neurological symptoms and radiological features occurring ≥ 3 months after the initial ADEM episode. • Commonly linked to the presence of anti-MOG antibodies.	• Prognosis ranges from moderate to favorable, with an elevated risk of chronic neurological deficits. • May require continued immunotherapy.	[5]
**ADEM with Optic Neuritis (ADEM-ON)**	• Variant of multiphasic ADEM characterized by the onset of optic neuritis after an initial ADEM event. • Frequently anti-MOG positive and primarily affects children.	• Prognosis remains uncertain, as patients experience multiple relapses and lasting vision and cognition deficits. • Often require long-term immunosuppression.	[135]
**Acute Hemorrhagic Leukoencephalitis (AHL/AHEM)**	• A rapidly progressive, severe form of ADEM, also known as Weston-Hurst syndrome, defined by brain edema, hemorrhage and necrosis.	• High risk of poor outcome, with a high fatality rate (~47%). • Long-term survivors typically suffer from severe and permanent neurological damage.	[136]
**Tumefactive ADEM**	• Typically presents with an acute or subacute onset, usually monophasic. • The disease course may appear severe because of mass effect and edema. • Often mimics a neoplastic process in MRI.	• Overall prognosis is favorable with appropriate treatment (high—dose steroids). • Long-term outcomes are generally good.	[122]

## Data Availability

No new data were created or analyzed in this study. Data sharing is not applicable to this article.

## Abbreviations

The following abbreviations are used in this manuscript:

ACE2	Angiotensin-converting enzyme 2
ADCC	Antibody-dependent cell-mediated cytotoxicity
ADEM	Acute disseminated encephalomyelitis
ADEM-ON	Acute disseminated encephalomyelitis with optic neuritis
ADS	Acquired demyelinating syndromes
AHL/AHEM	Acute hemorrhagic leukoencephalitis
AI	Artificial intelligence
ALL	Acute lymphoblastic leukemia
AML	Acute myeloid leukemia
BBB	Blood–brain barrier
BMT	Bone marrow transplantation
Cho	Choline
CLL	Chronic lymphocytic leukemia
CMV	Cytomegalovirus
CNS	Central nervous system
CSF	Cerebrospinal fluid
DENV	Dengue virus
EBV	Epstein–Barr virus
EEG	Electroencephalogram
HCV	Hepatitis C virus
HHV-6	Human herpesvirus 6
HIV	Human immunodeficiency virus
HLA	Human leukocyte antigen
HSE	Herpes simplex encephalitis
HSV	Herpes simplex virus
ICAM-1	Intercellular adhesion molecule-1
ICU	Intensive care unit
IL	Interleukin
IFN-γ	Interferon-γ
IVIG	Intravenous immunoglobulin
MBP	Myelin basic protein
MMP-9	Matrix metalloproteinase-9
MOBP	Myelin-associated oligodendrocyte basic protein
MOG	Myelin oligodendrocyte glycoprotein
MOGAD	Myelin oligodendrocyte glycoprotein antibody-associated disease
MRI	Magnetic resonance imaging
MRS	Magnetic resonance spectroscopy
MS	Multiple sclerosis
MuV	Mumps virus
MV	Measles virus
NAA	N-acetylaspartate
NMO	Neuromyelitis optica
OCT	Optical coherence tomography
OSP	Oligodendrocyte-specific protein
p-Tau	Phosphorylated Tau
PE	Plasma exchange
PLED	Periodic lateralized epileptiform discharge
PLP	Proteolipid protein
PMNS	Post-malaria neurological syndrome
PBSCT	Peripheral blood stem cell transplant
SARS-CoV-2	Severe acute respiratory syndrome coronavirus 2
TDLs	Tumefactive demyelinating lesions
TGF-β	Transforming growth factor-β
TIMP-1	Tissue metallopeptidase inhibitor-1
TNF-α	Tumor necrosis factor-α
VCAM-1	Vascular cell-adhesion molecule-1
VZV	Varicella zoster virus
